# Evaluation of Residual Stress Distribution and Relaxation on In Situ TiB_2_/7050 Al Composites

**DOI:** 10.3390/ma11050706

**Published:** 2018-04-30

**Authors:** Kunyang Lin, Wenhu Wang, Ruisong Jiang, Yifeng Xiong, Dezhong Zhao

**Affiliations:** The Key Laboratory of Contemporary Design and Integrated Manufacturing Technology, Ministry of Education, Northwestern Polytechnical University, Xi’an 710072, China; linkunyang@mail.nwpu.edu.cn (K.L.); npuwwh@nwpu.edu.cn (W.W.); xiongyifeng@mail.nwpu.edu.cn (Y.X.); zhaodz@mail.nwpu.edu.cn (D.Z.)

**Keywords:** residual stress, metal matrix composites, contour method

## Abstract

Interior residual stresses induced by quenching may cause distortion during subsequent machining processes. Hence, various strategies have been employed to relieve the interior residual stress, such as stretching, post treatment, and other techniques. In this study, the stress distribution inside TiB_2_/7050 Al composite extrusions was investigated and the effects of different methods on relieving the quenching-induced stress were compared. Firstly, three TiB_2_/7050 Al composite extrusions were treated by stretching, stretching and heat treatment, and stretching and cold treatment processes, respectively. Then, the multiple-cut contour method was employed to assess the residual stresses in the three workpieces. Experimental results indicate that the interior stress of TiB_2_/7050 Al composite extrusions after stretching ranges from −89 MPa to +55 MPa, which is larger than that in 7050 aluminum alloy, which ranges from −25 Pa to +25 MPa. The heat treatment performs better than the cold treatment to reduce the post-stretching residual stress, with a reduction of 23.2–46.4% compared to 11.3–40.8%, respectively. From the stress map, it is found that the stress distribution after the heat treatment is more uniform compared with that after the cold treatment.

## 1. Introduction

Particle-reinforced metal matrix composites (PRMMCs) have received intensive attention due to their excellent properties—such as low density, high strength and modulus, improved thermal stability, and increased wear resistance—which make them potential candidate materials in aerospace, automobiles, and numerous other fields [1,2,3]. Like other metallic materials, solution treatment and quenching are employed to enhance the PRMMCs’ properties. In these processes, a high temperature field and severe temperature gradient are inevitable, resulting in a high interior residual stress field in the raw materials. Residual stresses may induce premature failure through cracking, reduced fatigue strength, induced stress corrosion, or hydrogen cracking, and cause distortion and dimensional variation [4,5,6,7].

Robinson et al. [8] measured the through-thickness residual stress distribution in 7449 aluminum alloy-forging by neutron diffraction and the deep hole drilling method. They reported a large magnitude (>250 MPa) tensile residual stress in the center of an as-quenched forging and compression residual stress (< −200 MPa) in the surface region. In order to reduce the residual stress without affecting the material properties, different methods surrounding the parts, like changing the thermal gradient and mechanical means, have been attempted and reviewed [9]. The stretching and cold compression methods are known to be very effective in reducing residual stress, according to both production and experimental results. In the report by Prime and Hill [10], both rolling direction and transversal direction residual stresses in 7050 aluminum alloy after the stretching process were reduced to the ±25 MPa range. Zhang et al. [11] found that the cold-compression process can relieve 43–79% peak stress magnitude in big T-shaped 7050-T7452 aluminum alloy forgings. However, these processes cannot relieve all interior stresses, generally. The remaining stress always leads to part distortion or dimensional instability during subsequent machining processes. In the workshop, post-treatment is employed to relieve the remaining stress after rough machining. Unfortunately, these processes are always done by experience. Hence, accurate measurements of the magnitude and distribution of stress state to direct a residual stress relief process is deemed necessary.

Residual stress analysis is divided into nondestructive techniques (e.g., laboratory X-ray diffraction, neutron diffraction, synchrotron X-ray diffraction), semi destructive techniques (e.g., layer removal method, drilling method), and destructive technique (e.g., crack compliance method, contour method) [12,13]. Among all these methods, the layer removal method and crack compliance method can only measure a 1D stress profile. The deep hole drilling method has limited spatial resolution. X-ray diffraction can only measure near surface residual stresses. Neutron diffraction and synchrotron X-ray diffraction are powerful methods, which can be used to map multiple residual stress components in relatively thick bodies. However, both neutron diffraction and synchrotron X-ray diffraction require highly specialized facilities, which are expensive to operate and have limited availability. The contour method is a new destructive method invented by Prime based on Bueckner’s superposition principle in 2001 [14]. The superiority of the contour method is in providing a two-dimensional map of residual stresses that are typical of a cut plane of interest, with high accuracy compared with other residual stress measurement methods. In the past decade, due to the advantages of simple, cost effective, and little need for specialized equipment, the contour method has been widely used in academic research and engineering applications [15,16,17,18,19]. Combining other residual stress measurement technologies, enhanced contour methods have also been developed to measure more stress components [20,21,22,23,24].

The residual stress states in different kinds of aluminum alloys have been widely studied [8,10,11]. However, the investigations on PRMMCs have focused on studying the machinability of tool wear, surface integrity, and chip formation [25,26]. Few reports have focused on the machining distortion caused by residual stress inside PRMMCs. On the other hand, quantitative investigations of the residual stress state in PRMMCs and the effects of different post-treatments for reducing the post-stretching residual stress in PRMMCs are scarce, so far. In this work, three in situ TiB_2_/7050 Al composite extrusions with the same production process and geometry were prepared by an in situ synthesis technique. One was left in the as-quenched condition, while the other two underwent post-treatment by applying either heat or cold treatments. The multiple-cut contour method was used to measure the interior residual stresses of all extrusion components. The residual stress distributions on the same cut plane in the extrusion components were compared and analyzed. The effects of stretching and two post-treatment processes on reducing residual stress were also investigated. The objectives of this investigation are: (a) to quantify the residual stress in rectilinear extrusions made from TiB_2_ particle-reinforced aluminum matrix composites; (b) to investigate the available processes for relieving interior stresses.

## 2. Preparation of Test Components

### 2.1. Materials

**Figure 1:**
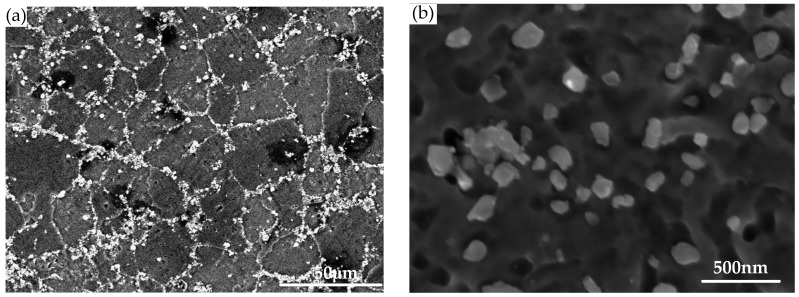
Microstructure of TiB_2_/7050 Al composite: (**a**) SEM image shows the distribution of TiB_2_ particles; (**b**) magnified TiB_2_ particles aggregated along the grain boundaries.

The PRMMCs used in this study were in situ 6 wt % TiB_2_ particle-reinforced 7050 aluminum matrix composites (referred to as TiB_2_/7050 Al composites), which were prepared by the State Key Laboratory of Metal Matrix Composites of China [2]. The TiB_2_/7050 Al composites were synthesized via the controllable salt–metal reaction technique via mixed salts of K_2_TiF_6_ and KBF_4_ [27]. They were homogenized at 470 °C for 24 h and then extruded. The solution treatment was carried out at 477 °C for 190 min followed by a water quenching process. In order to lower the quenching stress, 1.5% prestretching was performed along the longitudinal direction. Afterwards, artificial aging treatment was carried out on the composite at 120 °C for 20 h. The nominal chemical composition of TiB_2_/7050 Al composite is shown in Table 1. The mechanical and physical properties of TiB_2_/7050 Al composite is shown in Table 2. The cross-sectional microstructure of the composite is depicted in Figure 1. With the in situ synthesis technique, the reinforcement particles are uniformly distributed in the matrix and the reinforcement–matrix interfaces are clean [28]. The reinforcement particles are of a fine size, ranging from 20 to 500 nm [29]. In this study, three in situ TiB_2_/7050 Al composite extrusion blocks with the same dimension of 169.7 mm (longitudinal direction) * 84.4 mm (longitudinal-transversal direction) * 64.4 mm (short-transversal direction) were used.

### 2.2. The Post-Treatment

**Figure 2:**
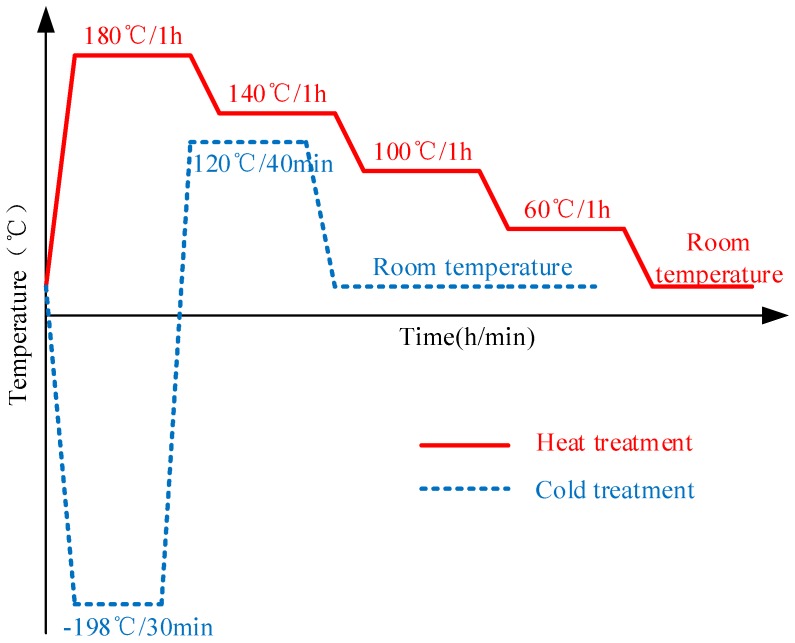
The processes of heat and cold post-treatment.

Of the three specimens, the first one was left in the as-quenched condition, as mentioned above, while the other two experienced post-treatment by applying either heat or cold treatment. The processes used for heat and cold treatment are illustrated in Figure 2. The second specimen was under heat treatment. Heat treatment was performed in a furnace with initial temperature of 180 °C. After 1 h of heat preservation, the furnace temperature was lowered by 40 °C step-by-step (1 h per step) down to room temperature. The third specimen underwent cold treatment: it was immersed in liquid nitrogen (−198 °C) for 30 min, then quickly transferred into a 120 °C furnace, remaining there for 40 min, and, lastly, cooled inside the furnace to room temperature.

## 3. Residual Stress Measurement

### 3.1. Basic Principle for Multiple-Cut Contour Method

Compared with the basic contour method [14], the multiple-cut contour method provides the advantage of measuring multiple stress components on different cut planes. To comprehensively assess the residual stress in the in situ TiB_2_/7050 Al composites with different stress reduction treatments, the multiple-cut contour method was chosen. The principle of multiple-cut contour method will be briefly reviewed below [30].

**Figure 3:**
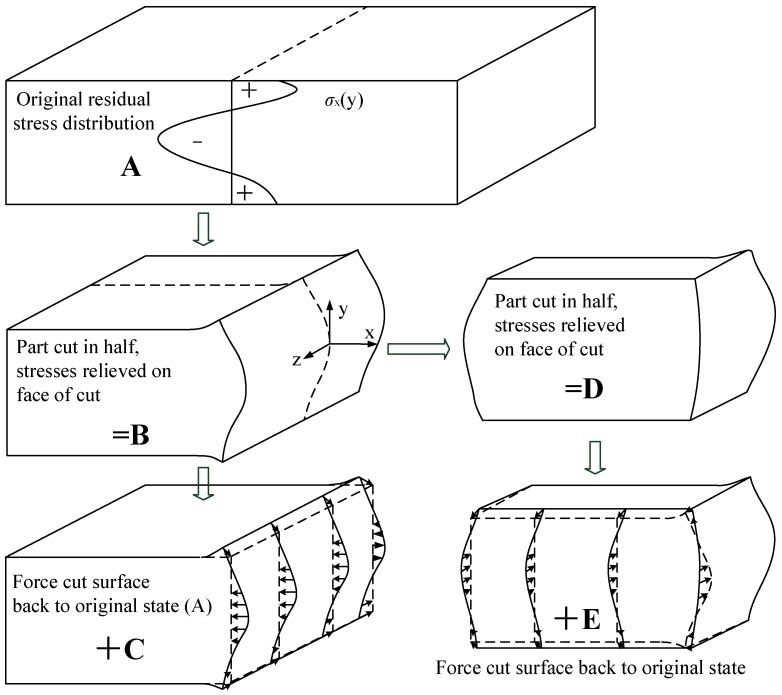
Contour method superposition principle for traditional contour method (**A**–**C**) and multiple cuts (**D**–**E**). The two-cut plane defines *x* = 0 and *z* = 0 [28].

Figure 3 shows the basic contour method in steps A–C. Assuming elasticity, superimposing the partially relaxed stress in B with the change in stress from C gives the original residual stress throughout the part:

(1) $$\sigma^{\left(A\right)}(x,y,z)=\sigma^{\left(B\right)}(x,y,z)+\sigma^{\left(C\right)}(x,y,z)$$

where *σ* without subscripts refers to the entire stress tensor. The normal (*σ*) and shear (*τ*) stresses acting on the free surfaces in *B*, *σ_z_*, *τ_zx_*, and *τ_zy_* must be zero. Therefore, *C* by itself gives those stresses along the plane of the cut:

(2) $$\begin{array}{l} \sigma_{z}^{\left(A\right)}(0,y,z)=\sigma_{z}^{\left(C\right)}(0,y,z)\\ \tau_{zx}^{\left(A\right)}(0,y,z)=\tau_{zx}^{\left(C\right)}(0,y,z)\\ \tau_{zy}^{\left(A\right)}(0,y,z)=\tau_{zy}^{\left(C\right)}(0,y,z) \end{array}$$

The part in *B*, which is half of the original part, is cut on the plane *z* = 0. However, due to the local stress relaxation caused by the first cut, the remained stresses are not the original stresses. **D** shows the quarter-part, after the cut on plane 2, deformed by the residual stress relaxation. *E* is an analytical step in which the surface created by the second cut is forced back to its flat shape before the second cut. The stress state in *B* is given by superimposing the stress in *D* with the change in stress form *E*. The original residual stress throughout the part in *A* is therefore given by the sum of the stress state in *D*, *E*, and *C*:

(3) $$\begin{array}{cc} \sigma^{\left(A\right)}(x,y,z) & =\sigma^{\left(B\right)}(x,y,z)+\sigma^{\left(C\right)}(x,y,z)\\ & =\sigma^{\left(D\right)}(x,y,z)+\sigma^{\left(E\right)}(x,y,z)+\sigma^{\left(C\right)}(x,y,z) \end{array}$$

The normal (*σ*) and shear (*τ*) stresses acting on the free surfaces in *D*, *σ_z_*, *τ_zx_*, and *τ_zy_*, must be zero. Therefore, the sum of *E* and *C* will give the original stresses along the planes of the second cuts:

(4) $$\begin{array}{l} \sigma_{x}^{A}(x,y,0)=\sigma_{x}^{E}(x,y,0)+\sigma_{x}^{\left(C\right)}(x,y,0)\\ \tau_{xz}^{A}(x,y,0)=\sigma_{xz}^{E}(x,y,0)+\sigma_{xz}^{\left(C\right)}(x,y,0)\\ \tau_{xy}^{A}(x,y,0)=\sigma_{xy}^{E}(x,y,0)+\sigma_{xy}^{\left(C\right)}(x,y,0) \end{array}$$

where $\sigma_{x}^{\left(C\right)}(x,y,0)$ have already been calculated from the finite element analysis (FEM) calculation of $\sigma_{z}^{\left(A\right)}(0,y,z)$. The same procedure can be applied to obtain the *σ_x_* component if the cut is made along the *y* = 0 plane.

### 3.2. Cutting of Specimen

**Figure 4:**
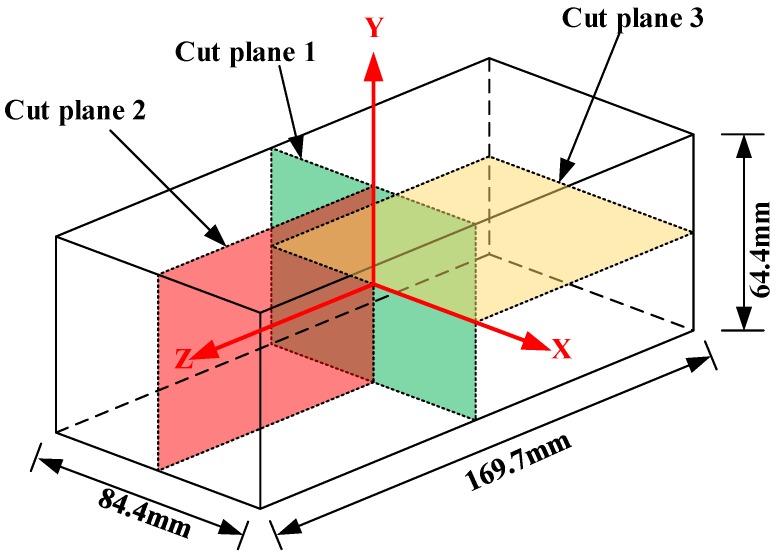
Dimensions of the TiB_2_/7050 Al composite extrusions and three-cut planes used to map residual stress in the extrusion.

**Figure 5:**
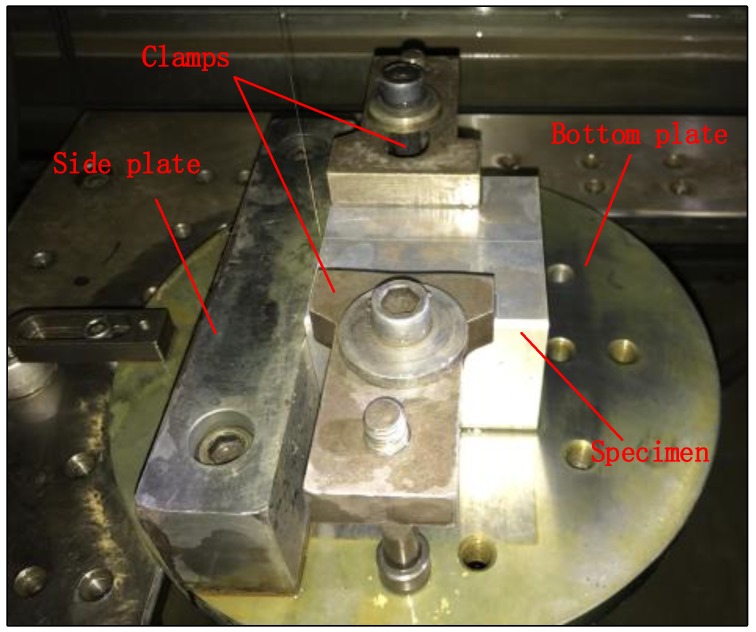
“L” shape fixture system.

The dimension of the blocks and cut locations are shown in Figure 4. The first cut along cut plane 1 sectioned the specimen into two halves and was used to measure the longitudinal residual stress. After the first cut, the contour of the surfaces created by the first cut on both halves was measured. Then, the cuts along cut plane 2 and 3 were made on the two halves in order to measures the long-transversal residual stress and short-transversal residual stress, respectively. In order to segment the specimen in halves without any plastic deformation, the wire electro discharge machining (WEDM) with skim cut mode and symmetry clamp is always suggested [14]. In this work, an “L” shape fixture system was used, as shown in Figure 5, to minimize the movement of the cut plane. Compared with the fixture system in other researches, the “L” shape fixture system can better restrain the thick block from moving and assure an accurate straight cut. Cutting was performed using an AgieCharmilles WEDM device (FI 240CC) with a 0.25 mm diameter hard brass wire at a cutting speed of approximately 0.6 mm/min. To prevent the thermal stress caused by the thermal imbalance during cutting, the specimens were immersed in deionized water tank to keep thermal equilibrium before cutting.

### 3.3. Surface Measurement and Data Processing

After cutting, all the specimens were left in the temperature-controlled measuring laboratory for 5–10 days to reach thermal equilibrium with the laboratory temperature. Then, the surface morphologies of both halves were scanned by a touch probe-based coordinate measuring machine (CMM) Leitz Reference Xi 1597. The surfaces were measured by continuous motion of the probe on the cut plane along the lines from one edge of the specimen to the opposite edge. A 0.5 mm × 0.5 mm grid density was used in the measurement process. The diameter of the CMM probe tip was 3 mm.

Although the WEDM cutting parameters and fixture system can lower the plastic deformation and limit the movement of the specimen, the inherent surface artefacts induced by cutting cannot be eliminated completely. Moreover, it is difficult to measure the deformation along the edges of the cut surfaces because the CMM probe tip may slip off the edges. Therefore, data processing is essential for an accurate evaluation of the stress. The main steps of data processing are as follows: removing the bad points; aligning the data for the two opposing surfaces; averaging the two opposing surfaces to remove shear stress effects and small cutting imperfections; and fitting the averaged data to an approximating function. In this study, the cubic spline-based algorithm, which is a common algorithm used in contour method, was used to fit the data with Matlab software [16,31].

### 3.4. FEM Calculation

Of the last step of contour method, an elastic finite element analysis was undertaken to calculate the original residual stress along the normal direction of the cut plane. The commercial software ABAQUS was used in this study. The half or quarter of the specimen was modeled in FEM with the same coordinate. The smoothed spline contour was applied to the FEM model as the displacement boundary condition, but with opposite sign. Three additional displacement constraints were applied at two corners in order to prevent the rigid body motion [14]. Due to the exothermic reaction process method, particles of TiB_2_/7050 Al composites are microsize and distribute uniformly in the base material. Thus, the TiB_2_/7050 Al composites are assumed isotropic in the FEM model. The Young’s modulus and Poisson’s ratio were 78 GPa and 0.33, respectively. The FEM model was meshed with linear hexahedral elements with reduction integration (C3D8R) with the size of 1 × 1 × 1 mm^3^. 

## 4. Results and Discussion

### 4.1. Residual Stress Distribution

**Figure 6:**
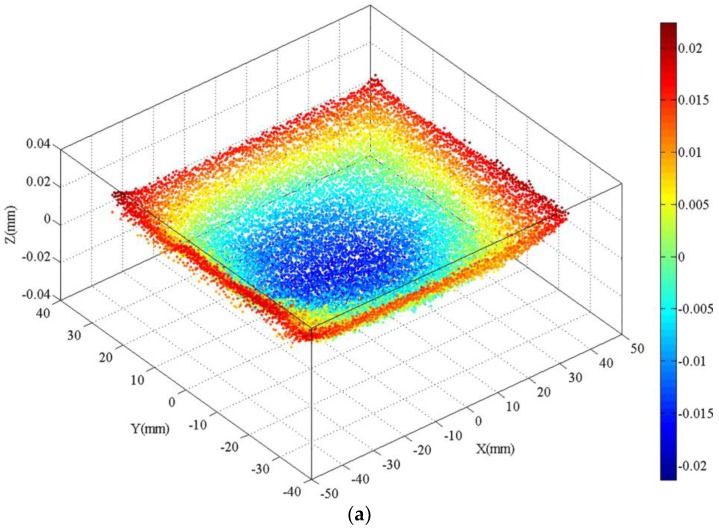

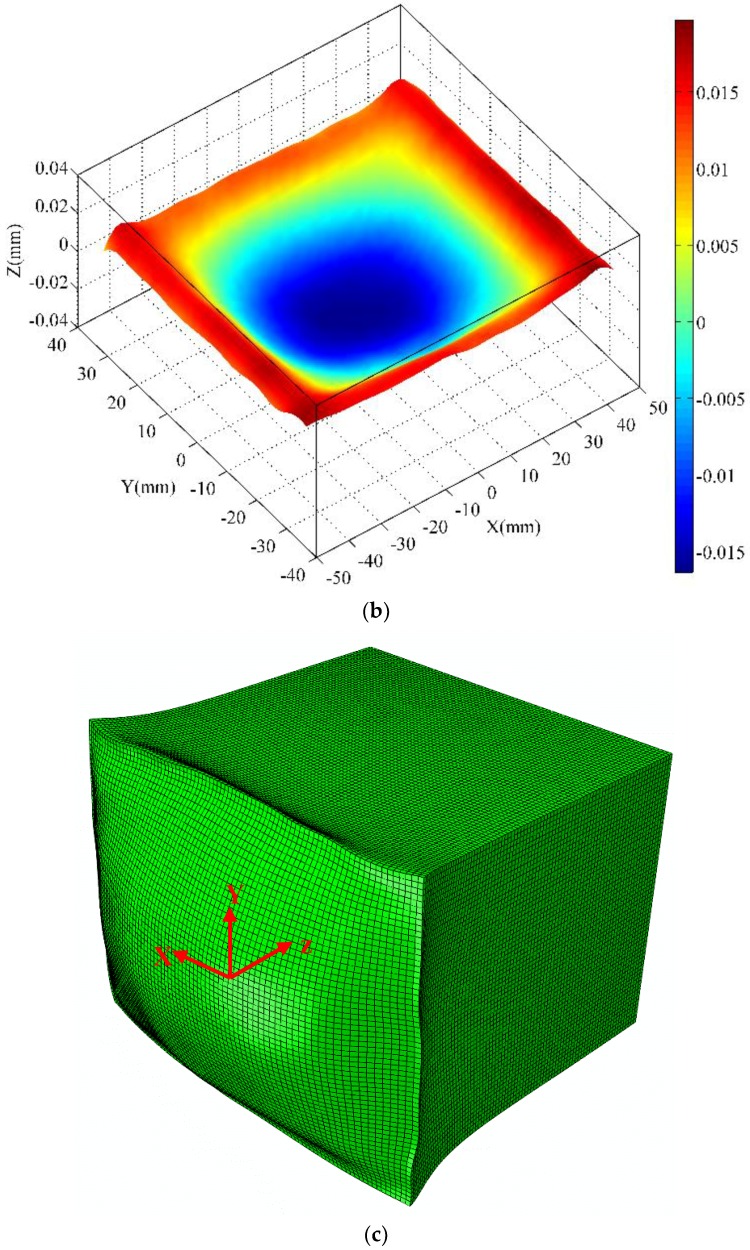
An example of coordinate measuring machine (CMM)-measured data-processing stages: (**a**) CMM-measured raw data for the cut plane 1 in the as-quenched specimen; (**b**) the smoothed surface for the cut plane 1 in the as-quenched specimen; (**c**) the 3D FEM model that applies the smoothed surface contour as the displacement boundary, with displacements magnified by a factor of 500.

The residual stresses of normal to three-cut planes were measured and mapped to assess the residual stress in the in situ TiB_2_/7050 Al composites and to compare the effects of different post-treatments on relieving residual stress. The cutting processes were stable without any artefacts such as wire break or chatter. The surface after cutting was smooth. Surface defects like cracks and holes, which often occur during machining of PRMMCs, were not seen on the WEDM surface. These results indicate that the contour method can be used for measuring the residual stress of in situ TiB_2_/7050 Al composites. In order to show the CMM-measured data-processing stages, an example of the raw data of the CMM measurement, smoothed surface, and 3D finite element model with the measured contour applied as the displacement boundary is shown in Figure 6.

**Figure 7:**
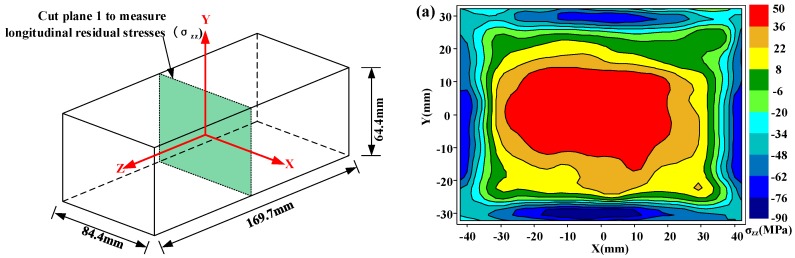

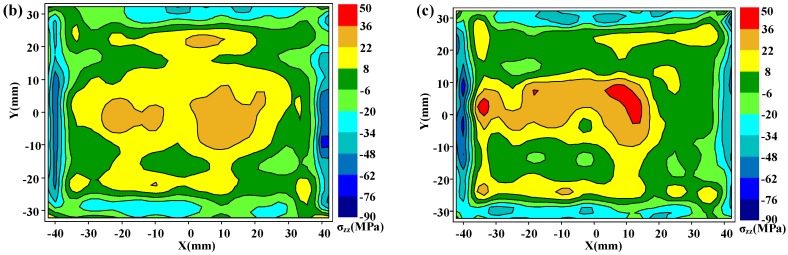
Longitudinal residual stresses (*σ_zz_*) on cut plane 1 in three different conditions: (**a**) as-quenched specimen; (**b**) heat-treated specimen; (**c**) cold-treated specimen.

It is known that residual stresses can be categorized into three types according to characteristic length scales: type I macrostresses, type II intergranular stresses, and type III atomic-scale stresses [13]. Although the intergranular stress (type II) in PRMMCs could be significant [32,33], it must be noted the residual stress measured here is type I of macrostress. Figure 7 shows the measured longitudinal residual stress (*σ_zz_*) maps on cut plane 1. In Figure 7a, the stress distribution pattern in the as-quenched specimen is approximately symmetrical with tensile stresses in the interior part and large compressive stresses along the boundary of the part, which has been reported in literatures [15]. As the prestretching is done in the material preparation stage, the magnitude of peak tensile stress (55 MPa) is lower. After heat and cold post-treatments, the measured longitudinal residual stress (*σ_zz_*) maps on cut plane 1 are presented in Figure 7b,c, respectively. Notable residual stress reduction can be seen in Figure 7b,c, but the residual stress distribution is not as symmetrical as the one without post-treatment. These asymmetrical distributions may be caused by the nonuniform or nonsymmetric thermal gradient in the warming or cooling process, as the specimens are thick.

**Figure 8:**
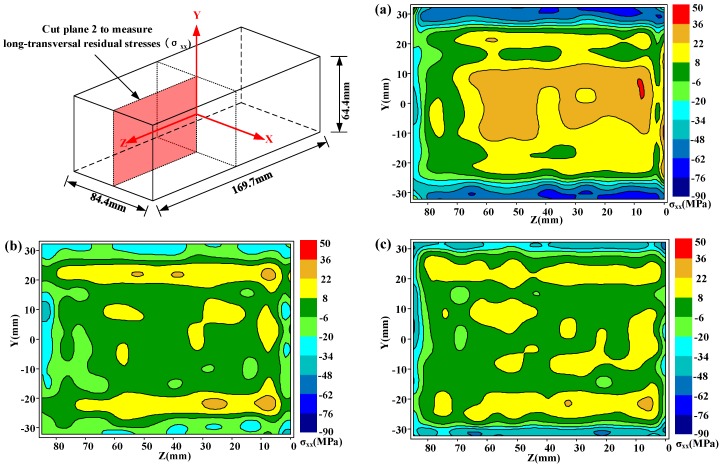
Long-transversal residual stresses (*σ_xx_*) on cut plane 2 in three different conditions: (**a**) as-quenched specimen; (**b**) heat-treated specimen; (**c**) cold-treated specimen. (The specific reconstruction process is omitted.)

**Figure 9:**
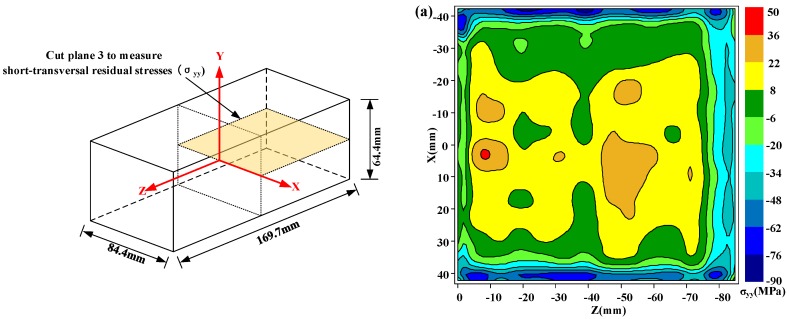

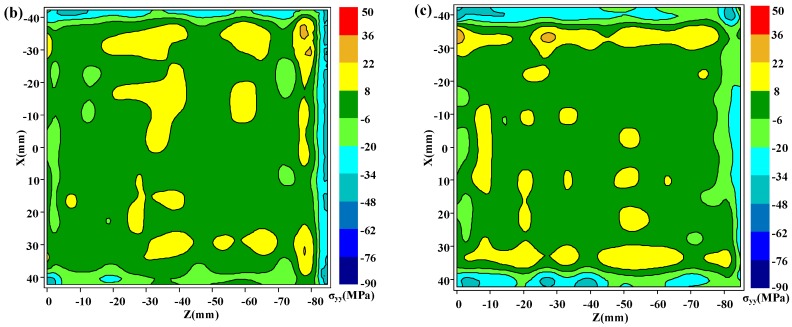
Short-transversal residual stresses (*σ_yy_*) on cut plane 3 in three different conditions: (**a**) as-quenched specimen; (**b**) heat-treated specimen; (**c**) cold-treated specimen. (The specific reconstruction process is omitted.)

Due to the first cut on plane 1, the stresses on cut plane 2 and cut plane 3 were locally released. Therefore, the original stresses on these two-cut planes must be reconstructed. According to the reconstruction process and principle referred to in the published research [16], which also have been reviewed in Section 3, the original stresses after construction are presented in Figure 8 and Figure 9. Figure 8 shows the original long-transversal residual stresses (*σ_xx_*) on cut plane 2 for the as-quenched, heat treatment, and cold treatment conditions, respectively. Figure 9 shows original short-transversal residual stresses (*σ_yy_*) on cut plane 3 for the same three conditions. Generally, the residual stresses on each cut plane show a similar distribution for the as-quenched state but of lower magnitude. As it is seen in these residual stress maps, the maximum tensile stresses are always at the center areas, whereas the compressive stresses are around the surface. In the water quenching process, a severe thermal gradient is always generated at the surface and the center of the part, leading to the formation of large compressive stresses at the surface and tensile stresses in the center of the part.

**Figure 10:**
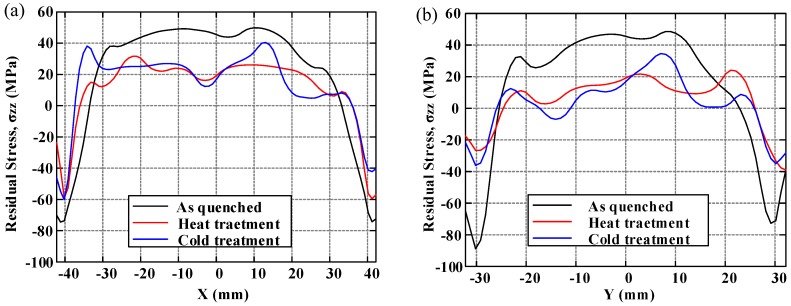
The distribution of longitudinal residual stress (*σ_zz_*): (**a**) along the *x*-direction at *y* = 0 mm; (**b**) along the *y*-direction at *x* = 0 mm.

**Figure 11:**
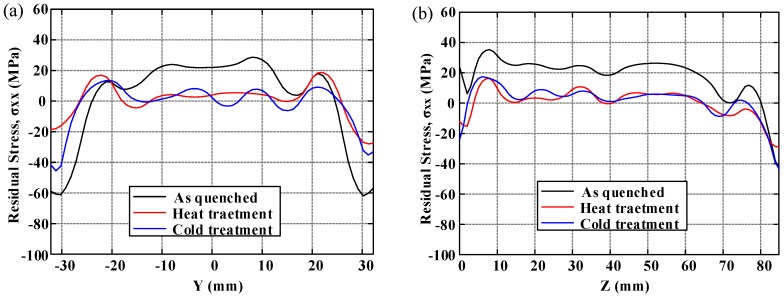
The distribution of long-transversal residual stress (*σ_xx_*): (**a**) along the *y*-direction at *z* = 42.4 mm; (**b**) along the *z*-direction at *y* = 0 mm.

**Figure 12:**
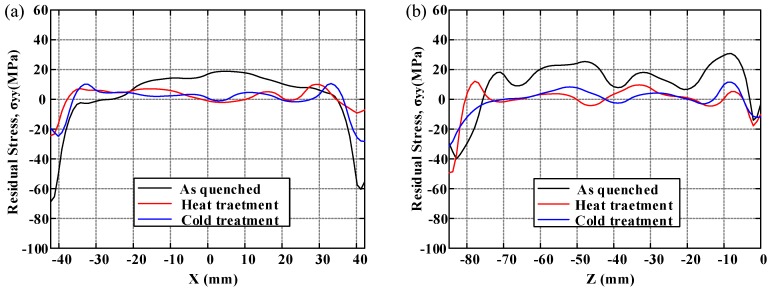
The distribution of short-transversal residual stress (*σ_yy_*): (**a**) along *x*-direction at *z* = −42.4 mm; (**b**) along *z*-direction at *x* = 0 mm.

In order to demonstrate the distribution of residual stresses and to compare the effect of residual stress reduction clearly under three conditions, the distributions of residual stress along two crossover lines on each cut plane are plotted. The longitudinal, long-transversal, and short-transversal residual stress distributions are shown in Figure 10, Figure 11 and Figure 12, respectively. The line plots show similar profiles—with compressive stress near the surfaces balanced by tensile stress in the center—but with different magnitude. These graphs clearly illustrate the reduction effect of post-treatment. Moreover, the magnitude of longitudinal stresses is larger than that of transversal residual stresses. These results would suggest that the geometry or thickness has significant effects on the residual stress distribution. The characteristics of local maxima and minima can be seen in these plots and have been reported by Prime and Hill [10]. They ascribe these characteristics to the through-thickness variation of yield strength caused by the crystallographic texture, composition gradients, and quench path.

### 4.2. Effect of Post-Treatment

**Figure 13:**
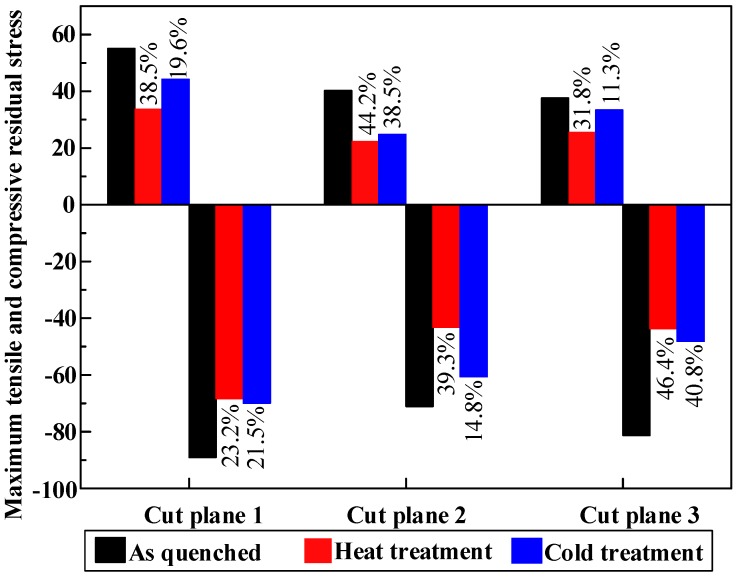
Maximum tensile (+) and compressive (−) residual stress in different cut planes with three treatment processes. The percentage means the maximum stress reduction of heat and cold post-treatment compared with the as-quenched specimen.

Stretching is the most effective mechanical method to reduce quenching residual stress [4]. This method has been reported in many researches and widely applied in industry [9]. In Reference [8], the residual stress of 7050 aluminum thick plate after water quenching ranges form −250 MPa to 200 MPa. After stretching, the residual stress has reduced to the range of ±25 MPa. It can be seen that stretching can relieve most of the residual stresses. The maximum tensile and compressive residual stress of TiB_2_/7050 Al composites in different cut planes with three treatment processes are illustrated in Figure 13. The residual stress of TiB_2_/7050 Al composites after stretching ranges from −89 MPa to +55 MPa in the as-quenched specimen, which is larger than the post-stretching residual stress in the matrix of aluminum alloy 7050. This is ascribed to the high strength of TiB_2_/7050 Al composites (630 MPa). During the quenching process, the thermal expansion mismatch between TiB_2_ and Al matrix at the interface leads to an increase in the internal stress and fairly high density of dislocations in the matrix of TiB_2_/7050 Al composites [34]. On the other hand, the uniform distribution of reinforcements coupled with good matrix-reinforcement interfacial integrity lead to high yield and ultimate strength. This is the strengthening mechanism of metal matrix composites [35]. The counterpart is that the high strength achieved by TiB_2_ particle reinforcement make the plastic deformation more difficult during the stretching process. It is inferred that the high strength and TiB_2_ reinforcement particles may hinder the stretching to relieve more stresses.

After the prestretching stress relief process, the peak magnitude of longitudinal (55 MPa tensile and −89 MPa compressive), long-transversal (36 MPa tensile and −61 MPa compressive), and short-transversal (34 MPa tensile and −75 MPa compressive) residual stresses on each direction are different. Clearly, the longitudinal stresses vertical to the prestretching direction are larger than the transversal stresses that are parallel to prestretching direction. This feature can also be seen in the residual stress distribution in each specimen. This difference indicates that the plastic deformation caused by stretching in the longitudinal direction is larger than that in the transversal direction. On the other hand, the result also quantitatively demonstrates that the triaxial residual stress state can be alleviated by uniaxial plastic deformation [9].

**Figure 14:**
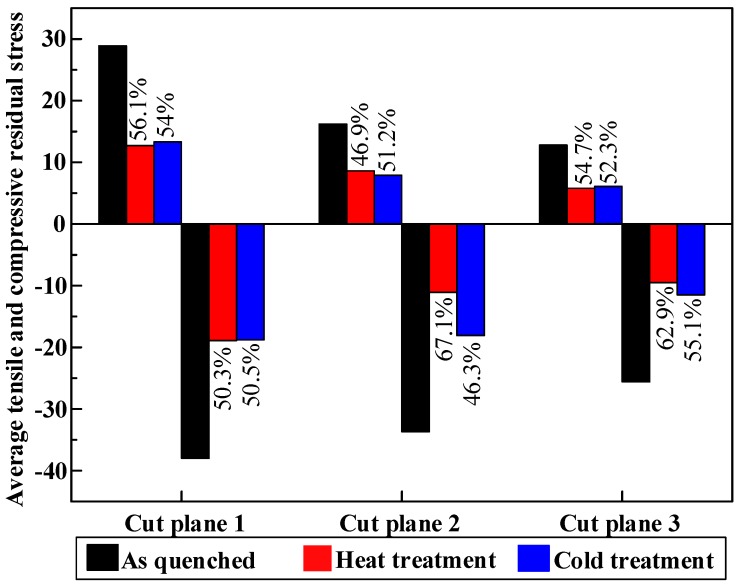
Average tensile (+) and compressive (−) residual stress in different cut planes with three treatment processes. The percentage means the maximum stress reduction of heat and cold post-treatment compared with as-quenched specimen.

As discussed previously, stretching can reduce most of the quenching-induced residual stresses but cannot remove all of them. The purpose of post-treatment is to relieve the remaining stresses. In Figure 13, obvious residual stress relief effects after post-treatment can be seen. Magnitudes of maximum tensile stresses are about 55 MPa, 34 MPa, and 44 MPa for the as-quenched specimen, the heat treatment specimen, and the cold treatment specimen, respectively. On the other hand, magnitudes of maximum compressive stresses are about −89 MPa, −68 MPa, and −70 MPa for the as-quenched specimen, the heat treatment specimen, and the cold treatment specimen, respectively. Based on the data, after cold treatment, around 11.3–40.8% reduction of post-stretching residual stresses is achieved. Heat treatment performs better to reduce the maximum stress magnitude, with a reduction of 23.2–46.4%. It must be noted that the reduction percentages were calculated using the maximum residual stress values. The average tensile or compressive residual stresses of each cut plane by different treatments were calculated by averaging the nodes with tensile or compressive stress in the FEM model and is shown in Figure 14. It can be seen more clearly that, after post-treatment, the residual stress is obviously relieved.

**Figure 15:**
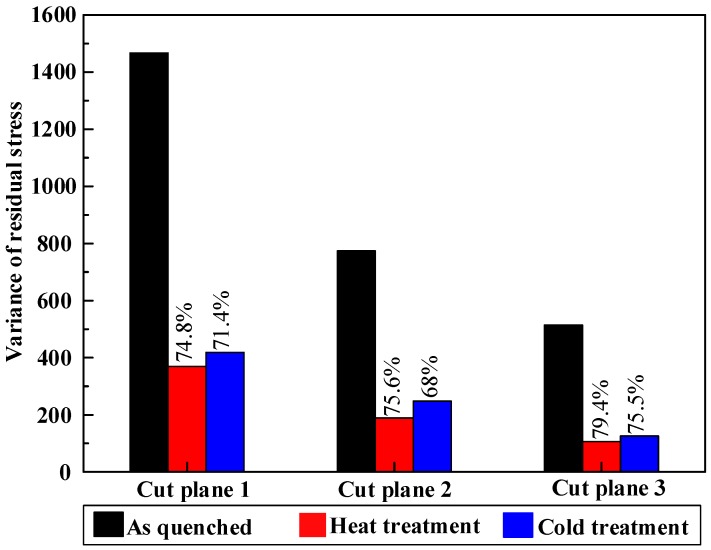
The variance of residual stress on each cut plane in different condition.

The variance of the measured residual stress on each cut plane is presented in Figure 15 to illustrate the stress distribution in different condition. The variance was calculated by Equation (5):

(5) $$VAR=\frac{\sum_{i=1}^{n}(\sigma_{i}-\bar{\sigma }{)}^{2}}{n}$$

where *n* represents the magnitude of node numbers on each cut plane in the last FEM calculation step of contour method measurement, $\sigma_{i}$ represents the stress on every node, $\bar{\sigma }$ represents the averaged residual stress on all the nodes. A lower *VAR* value implies a more uniform residual stress distribution. No matter the cut plane, the residual stress variance of different treatments, ranging from high to low, is as-quenched, cold treatment, and heat treatment. It can be concluded that heat treatment leads to a lower and more uniform stress distribution compared with cold treatment. However, both heat treatment and cold treatment still cannot relieve all the post-stretching residual stress. In order to reduce more residual stress due to quenching, an optimum process should be tried according to a different material property, quenching condition, and block geometry.

## 5. Conclusions

The interior residual stresses in three TiB_2_/7050 Al composite blocks were measured and mapped using the multiple-cut contour method. To reduce the post-stretching residual stress, two post-treatment processes (heat treatment and cold treatment) were used, and the effects were investigated and compared at the same cut planes. The main conclusions drawn are as follows:

(1) The multiple-cut contour method is suitable for measuring the internal stress for in situ TiB_2_ particle-reinforced aluminum matrix composites. The residual stress with small magnitude after stress relief can be assessed precisely by multiple-cut contour method.
(2) Due to the high strength of TiB_2_/7050 Al composites and the existence of TiB_2_ reinforcement, the interior residual stress after stretching ranges from −89 MPa to +55 MPa, which is larger than the residual stress magnitude inside 7050 alloy after stretching.
(3) The longitudinal residual stress parallel to the stretching direction is higher than the transversal residual stress perpendicular to stretching direction. This has also quantitatively demonstrated that uniaxial stretching can reduce the triaxial residual stress.
(4) Post-treatment is found to reduce the post-stretching residual stress further. The reduction of maximum residual stress magnitude by heat treatment and cold treatment is 23.2–46.4% and 11.3–40.8%, respectively. Moreover, the stress distribution after the heat treatment is more uniform compared with that after the cold treatment.

## Figures and Tables

**Table 1:** Nominal chemical composition of TiB_2_/7050 Al composite.

Elements	TiB_2_	Cu	Mg	Zn	Zr	Al
Content/wt %	6	2.2	2.3	6.3	0.11	Balance

**Table 2:** Mechanical and physical properties of TiB_2_/7050 Al composite at 20 °C.

Material	Density (g/cm^3^)	Yield Strength (MPa)	Ultimate Tensile Strength (MPa)	Elastic Modulus (GPa)	Elongation (%)
TiB2/7050	2.9	630	700	78	6

## Supplementary Materials

Supplementary File 1
